# Factors that influence consumer viewing behavior in large-scale preview screenings in Chinese film market

**DOI:** 10.1371/journal.pone.0315726

**Published:** 2024-12-31

**Authors:** Cong Shen, Xin Hao

**Affiliations:** 1 School of Management, Henan University of Technology, Zhengzhou, China; 2 Zhengzhou University of Aeronautics, Zhengzhou, China; Kaplan Business School of Australia, AUSTRALIA

## Abstract

The large-scale preview screenings during the summer season of 2023 hit box office records in the Chinese film industry. The rising box office earnings of widely distributed films indicate an increasing consumer propensity to watch movies in the post-pandemic period. Nevertheless, there is a lack of research about the consumption patterns associated with large-scale preview screening activity. This study examines the determinants of large-scale preview screening behavior by building a research model based on the theory of planned behavior. After interviewing 251 consumers from Zhengzhou, a newly selected first-tier city in China, we used Amos to analyze their patterns in attending large-scale preview screenings. According to our empirical study, consumers’ intention to watch movies on large-scale preview screening is positively and significantly affected by their perceived behavioral control, social network, and consumption expectation. Perceived behavioral control had the most significant influence, followed by social network and consumption expectation. These elements have a favorable and significant influence on consumers’ intention to watch movies. This study examines the main factors that influence consumers’ movie-watching habits and identifies the behavioral patterns that affect large-scale preview screening cinema attendance. The findings of this study can be a reference for increasing consumers’ passion for watching films. It offers vital recommendations for the recovery and sustainable growth of China’s film market in the post-pandemic period.

## 1. Introduction

Due to the market’s expansion and the severe rivalry, large-scale preview screenings have become a new marketing tactic in the Chinese film industry in recent years. Large-scale preview screenings refer to the practice of releasing a film before its general release to gauge audience reaction. Through a multitude of screening events and considerable media coverage prior to its official release, preview screenings not only raise audience’s expectations, but also generate hype for the official release [1]. China’s film industry has adopted a new strategy in promotion in the past two years. The summer box office in 2023 not only hit a record in Chinese movie history, but also raised the interests of the academic circle to study the large-scale preview screening. According to the statistics from China’s film industry, before their official release, a few of China’s summer holiday films for 2023 have already brought in hundreds of millions of RMB from the box office. For example, the ten-day preview screenings of Never Say Never helped the film earn 420 million RMB, which was the highest preview box office in history. According to data from Lighthouse, which is a professional film market research tool in China, No More Bets hit a record in ticket sales within a month. It accumulated 500 million RMB from all preview screenings. There is some debate about large-scale preview screenings, including worries about how they might affect the market and the viability of other films. However, in the post-pandemic era, large-scale preview screenings positively increase film visibility, collect audience feedback, bring in box office revenue, boost the film market and facilitate film promotion [2]. Therefore, it is important to study the consumer behavior under large-scale preview screening in order to understand the market demand, evaluate marketing strategy and improve the film market environment.

## 2. Literature review

There are many factors that affect consumers’ willingness to watch movies. When reviewing the research, it is found that elements besides the film affect customers’ decision to watch it, such as consumers’ personal traits and the surrounding environment. First, a number of elements can affect how people watch films, including film quality, reputation, entertainment value, brand integration, film type, creative team, cast, copyright, payment, movie-watching service, and so on [3–10]. These factors are reflected in how the film itself affects people. These studies show that different parts of the movie will have different levels of effect on viewers’ desire to watch movies. Secondly, apart from the impact of the film’s inherent attributes, consumers’ individual attributes also determine how much they want to watch films. Age, gender, educational attainment, socioeconomic level, and consumer psychology are factors that affect consumers’ desire to watch movies [11–15]. Some interesting research suggests that younger people might like science fiction and action movies, while middle-aged and older audiences might like drama or art movies. Higher-income consumers are more likely to pay for motion pictures. Some people might watch movies to unwind, find stimulation, or meet social responsibilities. Environmental factors also influence what people watch. Other factors, like the proximity of holidays, movie marketing and promotion tactics, fare concessions, and cinema environment can also affect consumer pleasure and willingness to watch movies [16, 17].

Recent studies have investigated consumers’ willingness to watch films through the lens of film attributes, consumer characteristics, and external environment. However, many studies start from a singular dimension. They fail to provide a systematic analysis of the factors that affect consumers’ willingness from a multidimensional integrated perspective. Large-scale preview screening of film presentation has distinct characteristics that differentiate it from traditional screenings, particularly in terms of screening duration, scale, version, audience demographics, and marketing strategies. Therefore, we need to examine factors that influence consumers’ large-scale preview screening behavior. These factors should include the film’s characteristics, consumer attributes, external environment, and others.

Distinct factors may influence customers’ decisions to go to the cinema, instead of choosing other forms of movie viewing, during large-scale preview screenings. It is important to discuss the key aspects that affect customers’ watching habits during the preview screening from their perspective. The event’s success will directly influence the distributor’s profitability and the overall vitality of the film industry. Consumers who attend large-scale preview screenings before formal release may have different viewing behaviors than typical customers. Film distributors must consider many things before extensive preview screenings, including their ability to transform preview box office income into ticket sales. Therefore, it is essential to understand consumer viewing intentions and behaviors during the cycle of preview screenings. To understand the regulations that control customer behavior during large-scale preview screenings, it is essential to understand what cause this phenomenon and how they affect consumers’ willingness to watch films. With this knowledge, we can make suggestions for the film and TV industry’s recovery from the epidemic and for its long-term growth. Consequently, from a multi-dimensional viewpoint, we built a 3D study model that includes those impacting consumers’ large-scale preview screening habit. This research examines the main factors that influence consumer viewing behavior in the new film exhibition model.

The innovation of this research is as follows: First, most research only looks at one part of the variables that influence moviegoers’ intentions to watch a film. In contrast to the theory of planned behavior, this study investigates multimodal latent factors that affect customers’ intentions to attend large-scale screenings. Secondly, this study has created a research model to identify key variables that influence moviegoers’ intention to watch movies. To verify the accuracy of this model, surveys were given to people who attended large-scale preview screenings. Based on the model of planned activity, we can explore key factors that influence consumers’ large-scale preview screening behavior and analyze specific situations in which planned behavior might be used. When combined with the viewing habits of consumers, the results of this study will help movie distributors and theaters create efficient film schedules that cater to the tastes of their customers. This will boost the on-demand screening business in China, which has been sluggish since the outbreak of covid-19. In 2023, when preview screening is still new in Chinese film industry, studying the movie-watching behaviors would help identify factors that influence consumers’ choice. With the knowledge of consumer psychology and consumer planning behavior, the scope of the study will be expanded.

## 3. Methods

### 3.1 Structural equation model and research hypotheses

**Fig 1:**
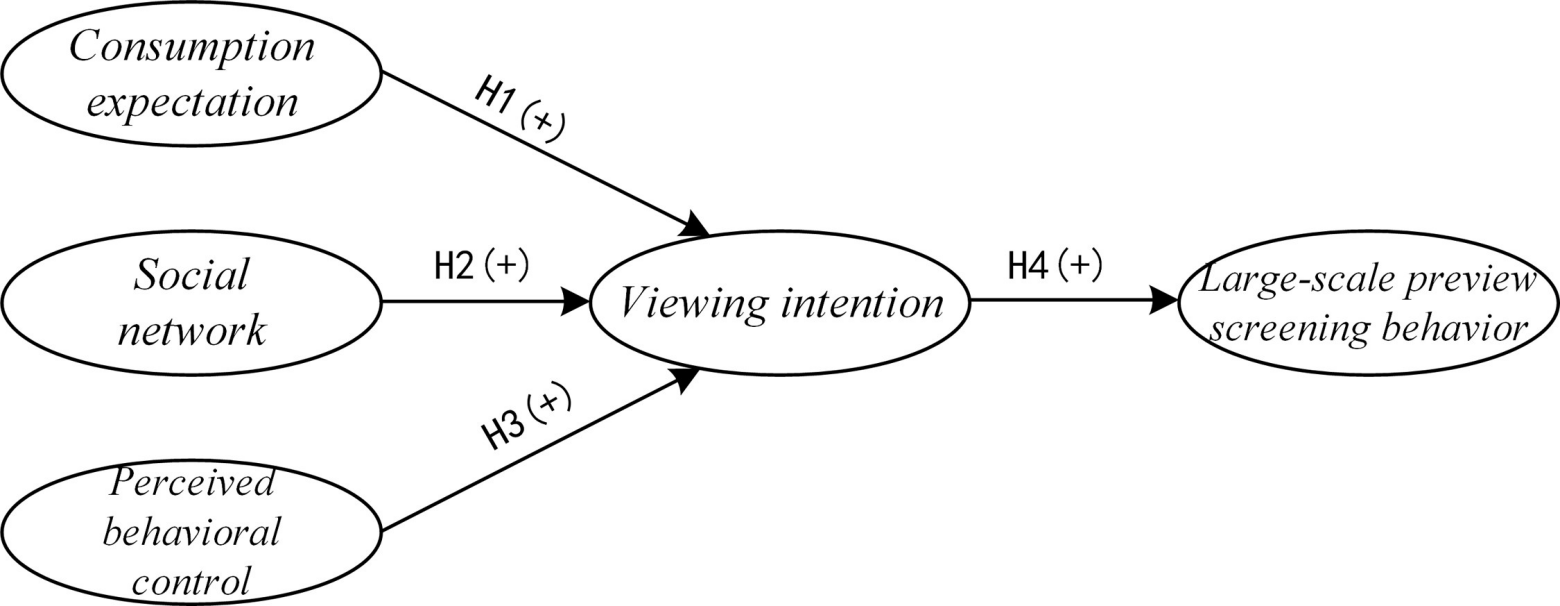
Research model.

The model of planned behavior provides a useful framework for understanding user psychology and the behavior it induced. It is used to study individual behavior in various contexts, especially the prediction of consumer behavior [18]. The theory of planned behavior (TPB) was proposed by professor Azjen [19] in 1985 as an extension of the rational behavior theory developed in 1980. TPB suggests that in the consumer market, consumer attitude, social network, and perceived behavioral control will influence consumer intentions and ultimately affect consumer behavior [20]. According to the theory of planned behavior, important people of the social network significantly affect consumer behavior. Consumers’ attitudes towards purchases are influenced by their view about the worth of their own consumption. Consumers’ decision-making and their perception of the purchase difficulty are related to their perceived behavioral control. The planned behavior model offers clear benefits when examining behavior influence and consumer psychology [21, 22]. Consumer psychological factors are more persuasive to explain consumers’ behaviors in large-scale preview screening. In this study, based on the theory of planned behavior, we constructed a research model for factors that influence consumers’ intentions to watch movies and their behaviors in the context of large-scale preview screenings. The model includes three-dimensional variables: consumption anticipation, social network, and the perceived behavioral control. The goal is to look at how these factors will influence consumer behaviors during large-scale preview screenings. Fig 1 shows the research model.

#### 3.1.1 Consumption expectation

Consumption expectation represents the subjective evaluations, feelings, or inclinations that consumers hold towards a particular product or service. Consumption expectation will affect their decision to attend or refuse to attend large-scale preview screenings. For instance, factors such as the ticket pricing strategies, the scheduling of film screenings, and the projection technology adopted by the cinema can all impact consumers’ decision to attend preview screenings [23]. Additionally, with the upgrade and optimization of cinemas in recent years, consumers’ intentions to watch movies are also affected by the services, the convenience of infrastructure, and the introduction of cultural elements [24, 25]. In this study, we defined consumption expectation as a key variable within the theory of planned behavior. Based on this, the following hypothesis is proposed.

H1: Consumption expectation has a significant positive impact on their viewing intention in the context of large-scale preview screenings.

#### 3.1.2 Social network

According to the theory of planned behavior, social network refers to whether most people’s opinions will influence an individual’s decision to engage in a behavior. Consumers’ intentions to watch movies are influenced by the social network they are in. In this study, we use social network to describe the existence of such a social network among potential consumers. In recent years, as internet technologies further advance, the influence of social network have increased. For example, many movie review websites have reduced the barriers of time and space, allowing potential consumers to make preliminary judgements about a film by reading reviews written by professionals [26–28]. Besides the influence of online technologies, the relationship between film recommenders and potential consumers also affects social network [29]. Some researchers have found that people are more likely to watch a movie if their close friends, family members, or coworkers with whom they have strong bonds also suggest it. The closer the relationship, the higher their viewing intentions will be. Professional judgment from people who do not share strong bonds can also moderately increase consumers’ viewing intentions as long as it can provide online social support [30–32]. In the preview screening market, where movies haven’t been released yet, a few movie critics who exhibit a deep knowledge about the industry are able to influence consumers intensions to watch the movies. Apart from that, most consumers are influenced by recommendations from professionals, media, events, friends and family members, and the word-of-mouth discussions. As consumers’ social network play an important role, the following hypothesis is proposed:

H2: Social network has a significant positive impact on consumer viewing intention in the context of large-scale preview screenings.

#### 3.1.3 Perceived behavioral control

According to the theory of planned behavior, perceived behavioral control refers to consumers’ cognitive perception of specific behaviors. Consumer’s perceived behavioral control over advance movie screenings is related to the film’s quality. Film quality, as expressed as word of mouth by fans and critics, is relevant to box office success. It also influences consumers’ viewing habits [33]. Current research on film quality mainly focuses on the characteristics of the film itself. For instance, Bian et al. proposed a three-level indicator system to evaluate film quality, including the film’s image, thought, and communicative competence [34]. Fu et al. and Liu found that factors such as sound effects, visuals, plot, and enjoyment all impact consumers’ evaluations of films and the word-of-mouth marketing [35–37]. Wang et al. discovered that the movie title design would influence whether a consumer accepts or rejects a film in different ways [38]. For example, for forward-looking films, textual titles can enhance the watching intentions, while titles with numbers are more effective in boosting the audience’s enthusiasm for sequels. The effects of word of mouth on sales are often delayed. Consumers have higher expectations and quality demands for film previews than the general release. Therefore, factors such as production costs, plot, and the director’s (personal) reputation become important indicators to predict potential consumers’ intentions to watch movies [8] and the following hypothesis is proposed:

H3: Perceived behavioral control has a significantly positive impact on consumer viewing intention in the context of large-scale preview screenings.

#### 3.1.4 Viewing intention

According to the theory of planned behavior, consumers’ intention to consume is the leading factor of consumers’ actual purchase as it affects consumer behavior. However, consumption intention is not equal to consumption behavior [39, 40]. This study suggests that consumers may attend large-scale preview screening when they intend to watch films. Therefore, we made the following assumptions:

H4: Viewing intention has a positive and significant impact on viewing behavior in large-scale preview screenings.

### 3.2 Research design

Structural equation modeling includes the structural model and the measurement model. The structural model, which measures the relationships between latent variables. In terms of research methodology, structural equation modelling has many advantages, such as the ability to work with multiple groups of dependent variables, to evaluate the model’s degree of fitting, and to calculate the relationship among different components.

We adopted random sampling methods to investigate viewers in Zhengzhou, a new first-tier city in China. The summer vacation of 2023 marks the first large-scale preview screening period in the Chinese film market. During this period, especially from July to August 2023, a lot of Chinese movies were released. To guarantee the authenticity of sample data, we randomly picked moviegoers who experienced large-scale previews as survey participants and sent them questionnaires offline. The survey procedure was reliable, and respondents had agreed to receive invitations to participate in questionnaires. We adopted random sampling to investigate a wide range of consumer groups in Zhengzhou. The advantages of random sampling of offline research include:

First, simple random sampling is a type of probability sampling in which the researcher randomly selects a subset of participants from a population. This easy-to-operate is crucial in statistical analysis and market research for its equal probability, unbiased random selection, broad application, robust theoretical base, and repeatability. In simple random sampling, every sample unit has an equal chance of selection. The sampling procedure is free from artificial bias or preconditions, hence guaranteeing the sample is a perfect representation of the population. As every sample unit has an equal chance of being selected, simple random sampling can lower sampling bias. Such sample data shows the attributes of the population data. In this way, the reliability of predictions can be improved. Basic random selection can yield acceptable sample data for either a community survey or a national study. Additionally, it works well with a variety of data, including numeric, subtyped, sequential data. Second, offline research collects data and feedback from target audience through traditional methods and channels. It is more accurate and reliable than online data. Compared to online survey, approaching people in person can yield higher response rates and facilitate face-to-face interactions, potentially leading to a more engaged audience. Furthermore, offline surveys often produce more detailed and nuanced feedback, since researchers may immediately answer to respondents’ questions, thereby improving the accuracy and depth of the data. These steps improve the study’s sampling methodology’s robustness and lessen selection bias even more.

Regarding to sample selection, we chose moviegoers in Zhengzhou, a Chinese new first-tier city. The participants were randomly selected. The sampling period was from July to August 2023, when large-scale preview screening first became popular in the Chinese film industry. The summer holiday of 2023 was considered as the earliest time of large-scale preview screening in China. To guarantee the reliability of the sample data collection, we randomly selected consumers with experience of preview screening to answer the survey. Before the survey was carried out, we obtained the consent of the respondents. The sampling method was random sampling, and the samples were from many consumer groups in China’s new first-tier city of Zhengzhou.

As the new first-tier city, Zhengzhou was selected in this research for the following reasons: First, Zhengzhou represents the emerging first-tier cities of China. Zhengzhou has more moviegoers than those in the second- and third-tier Chinese cities. Second, Zhengzhou is the capital of Henan, China. Located in northern Henan, it is one of the nine national central cities in China. The traits of Zhengzhou moviegoers may help explain the trends that the majority of Chinese moviegoers show. With a population of 12.8 million and a GDP of 1.29 trillion yuan, Zhengzhou became one of China’s 15 new first-tier cities in 2023. Currently, Zhengzhou has 135 theaters with almost 1,200 screens as well as top twelve box office receipts, solidifying its place in the film industry. Given its location, population, economy, and expanding film industry, Zhengzhou is designated as a model city.

The study underwent an ethical review, and informed consent was obtained before respondents completing the questionnaire. To protect personal privacy, each responder’s gender, age, income, and other relevant factors was kept secret during this survey. In this way, consumers are more likely to answer inquiries about their intentions to attend preview screenings. The questionnaire prioritizes measurement items as primary factors and demographic information as secondary variables. Likert scale, with 1 denoting "strongly disagree" and 7 denoting "strongly agree," was used to rate the primary variables. On a scale from 1 to 7, participants were asked to indicate their degree of agreement. The items on the questionnaire were taken from established literature and scales. Before the survey started, we conducted a pilot study and consulted experts to ensure the questionnaire’s quality.

## 4. Results and discussion

To assess the values’ reliability, our pilot study collects some questionnaires ahead of time and has specialists review the questions. Initially, we sent the questionnaire to experts for review and accepted their suggestions. Before investigation, we tested the validity and reliability of indicators and modified the questionnaire. After confirming the validity of the questionnaire, we distributed the questionnaires and collected the data. A total of 251 valid questionnaires were collected in the survey.

### 4.1 Population information statistics

**Fig 2:**
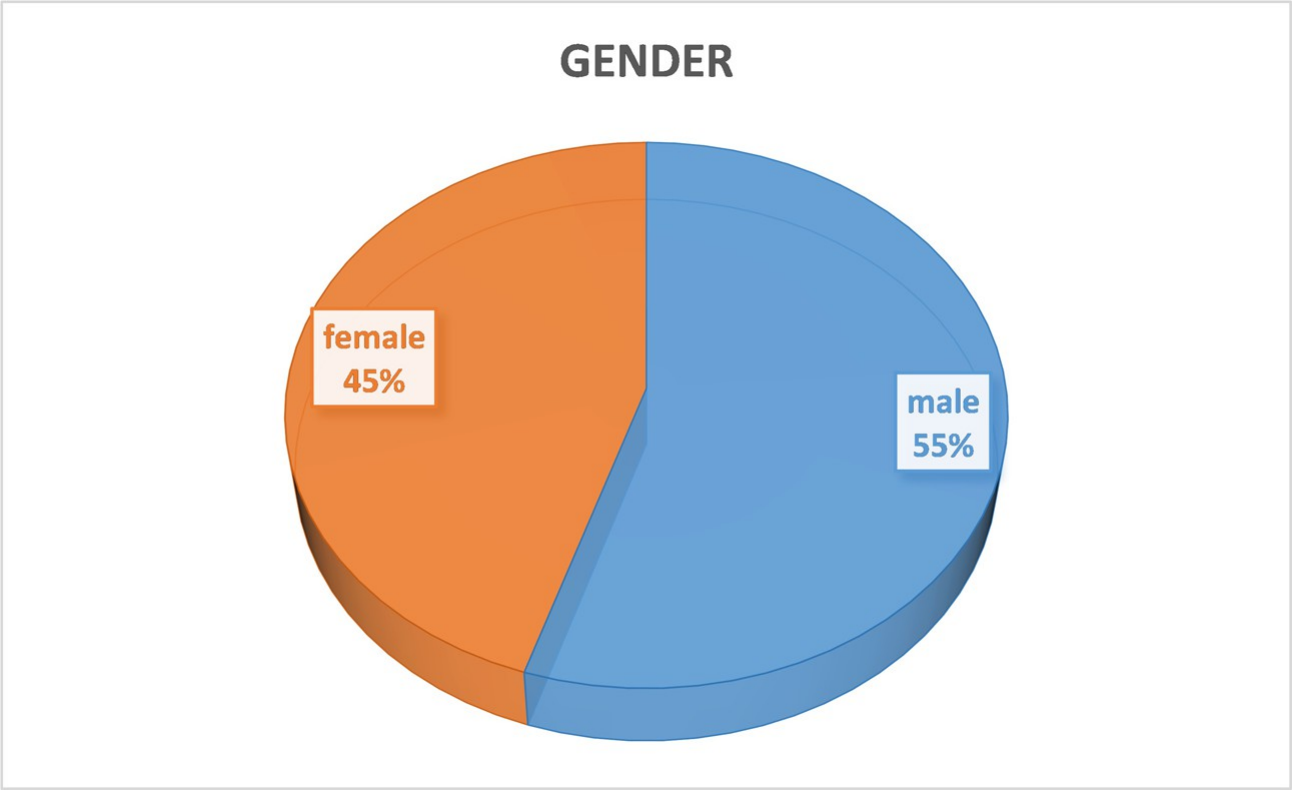
Gender of respondents.

**Fig 3:**
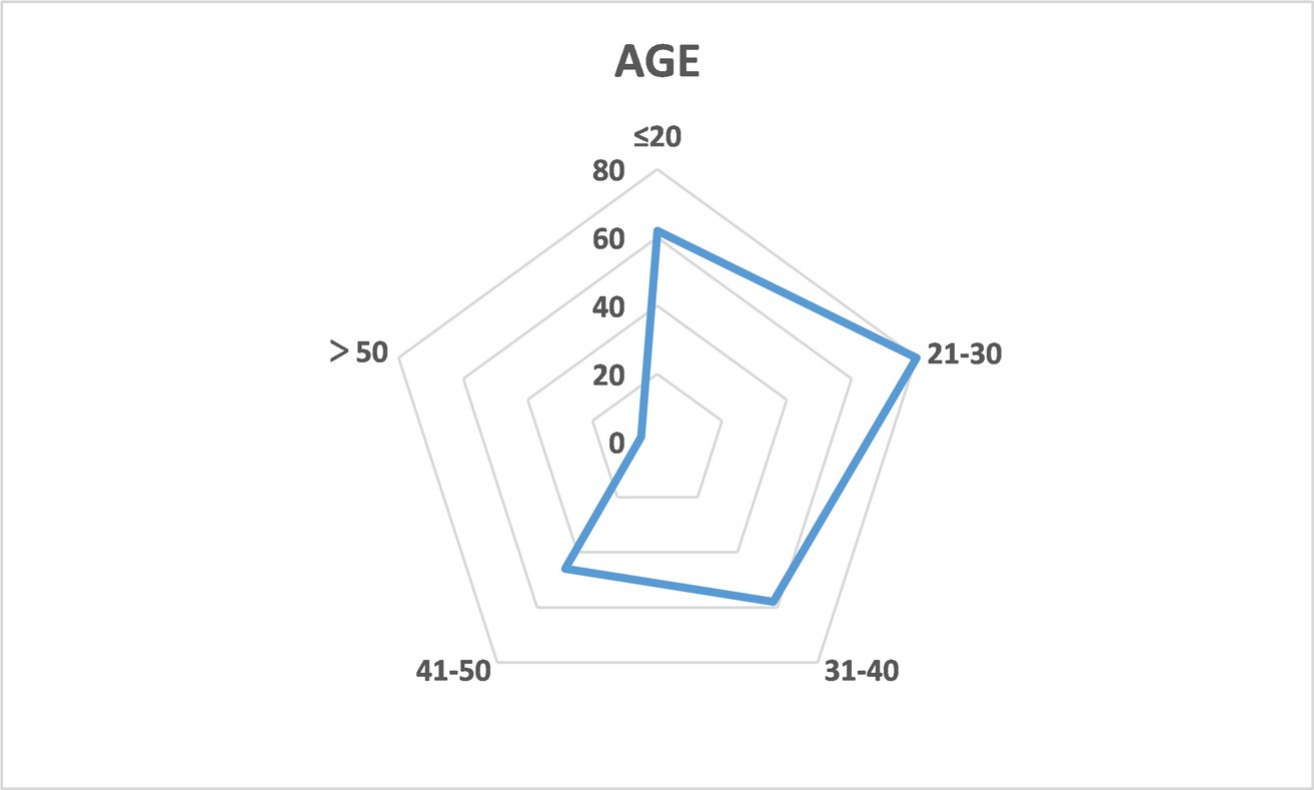
Age of the respondents.

**Fig 4:**
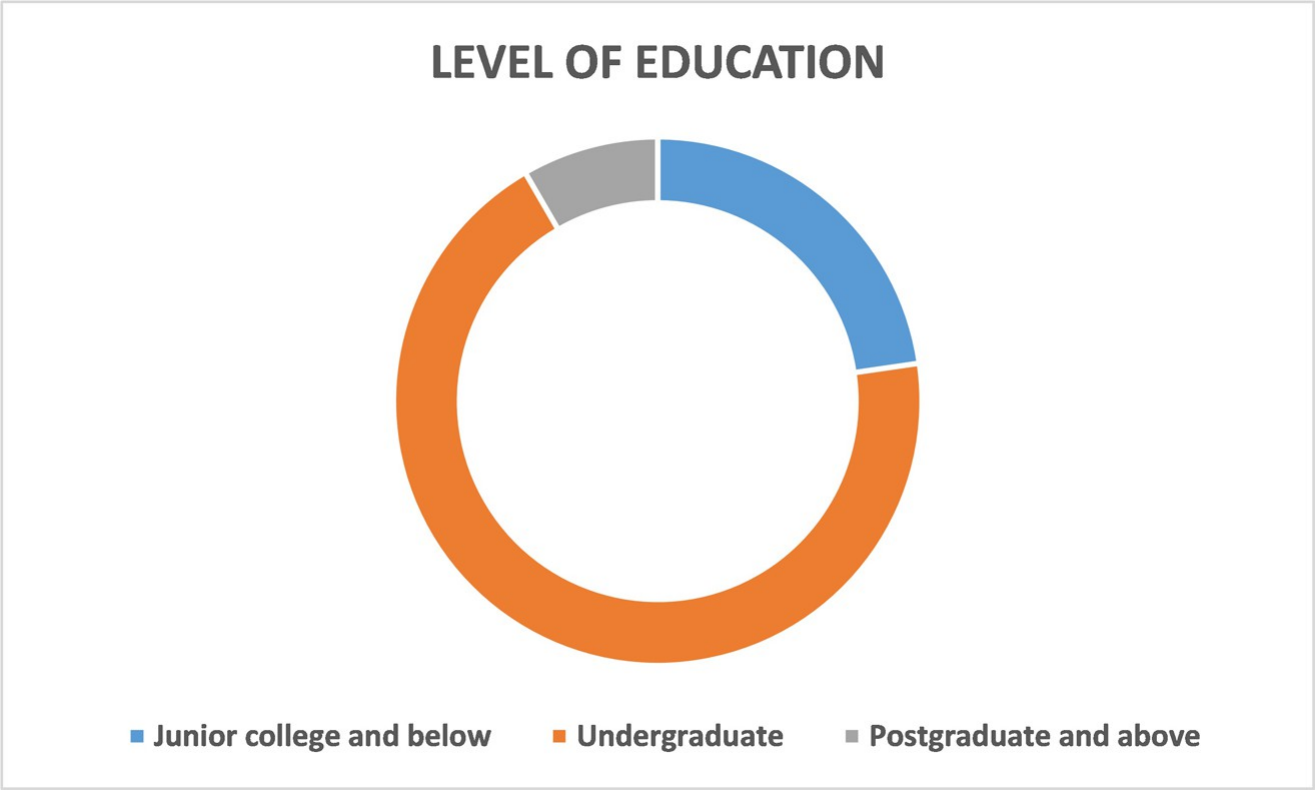
Education level of the respondents.

**Fig 5:**
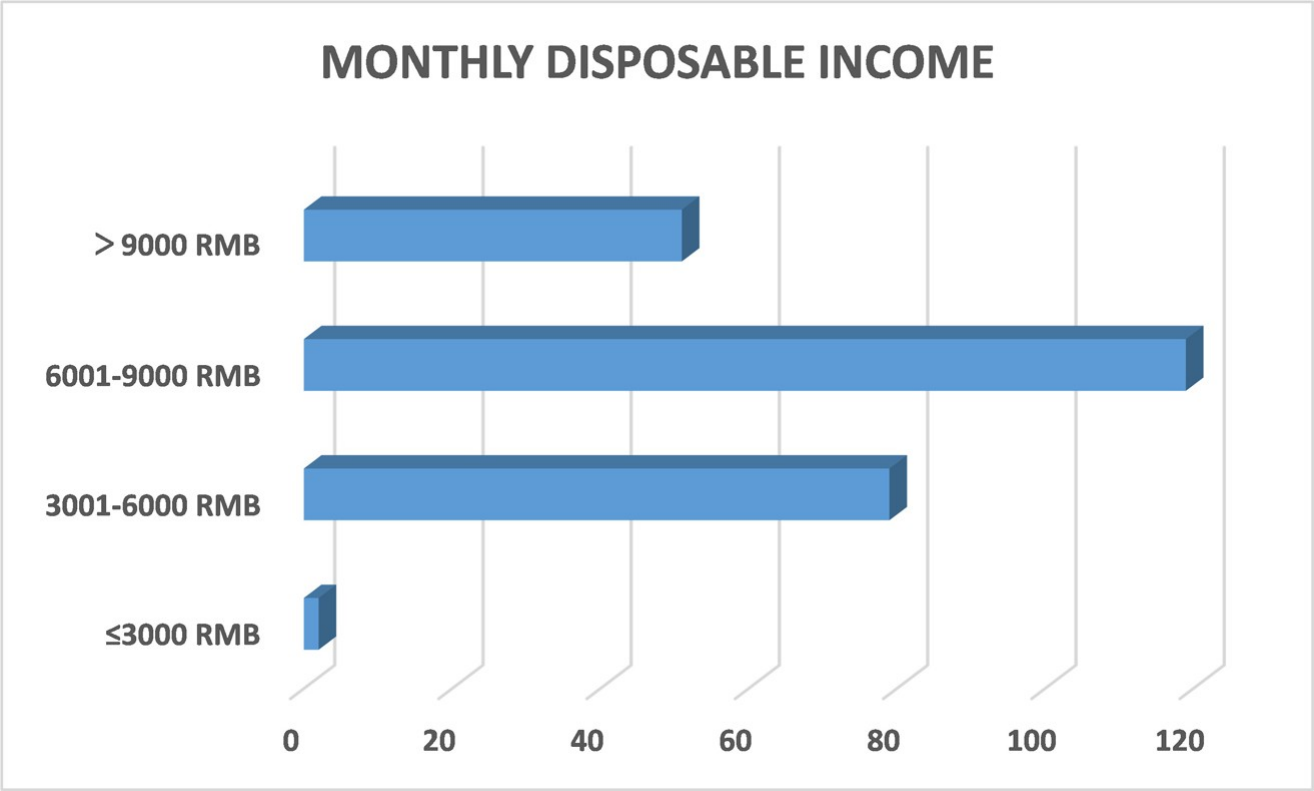
Statistics of average monthly disposable income of the respondents.

Statistics of respondents are shown from Figs 2–5, including their gender, age, education level, and disposable income. According to the demographic data, male respondents outnumbered female respondents by 10 percentage points. Nearly 80 percent of respondents were younger than 40 and more than one-third of them had an undergraduate degree. The average monthly disposable income of the respondents was over 3,000 RMB.

### 4.2 Empirical analysis

#### 4.2.1 Reliability and validity analysis

First, we evaluated the reliability and validity of the measuring scale. The model was evaluated using factor loadings, item reliability, and composite reliability. High factor loadings (close to 1 or -1) mean the factor strongly influences the variable; item reliability is the consistency of a set of items; and high composite reliability is a very good indication that all the items constantly measure the same construct. According to Table 1, the standard deviation values for factor loadings was over 0.6, signifying a robust correlation between each item and its corresponding factor. Moreover, the SMC values exceeded 0.36, indicating a strong internal consistency among the measurement items. The validity and composite reliability exceeded 0.7, showing a good consistency among the components of the observed variables. As the measuring scale demonstrated robust internal consistency and dependability, the accuracy of the measurements could be guaranteed [41].

**Table 1:** Design of measurement items and reliability analysis.

Dimensions	Items	Factor Loadings	Item Reliability	Composite Reliability	Reference
		STD.	SMC	CR	
**Perceived behavioral control (PBC)**	PBC1 The storyline is comprehensible.	0.681	0.464	0.722	Wang, 2023 [43]
	PBC2 No use of additional equipment to enhance the cost of the viewing experience.	0.655	0.429		
	PBC3 Possess a degree of familiarity with the director’s previous cinematic style	0.706	0.498		
**Consumption expectation (CE)**	CE1: I value whether the cinema is equipped with advanced screening technology	0.679	0.461	0.738	Chen et al., 2017 [24]
	CE2: I value whether the cinema schedules preview screenings during prime hours	0.679	0.461		
	CE3: I value whether the cinema can offer a fresh and advanced film-watching experience	0.728	0.530		
**Social network (SN)**	SN1: I would watch preview films recommended by film critics	0.705	0.497	0.692	Ma, 2024; Fu et al., 2020 [35, 44]
	SN2: My decision to go to the cinema is based on the advice of close friends who have viewed the firm earlier	0.627	0.393		
	SN3: I refer to the opinions of professional film review tools	0.629	0.396		
**Intention (INT)**	INT1: I am willing to go to the cinema to watch large-scale preview films.	0.639	0.408	0.693	Bian et al., 2015 [34]
	INT2: Large-scale preview screenings can attract me for film-watching.	0.650	0.423		
	INT3: I will continue watching large-scale preview films.	0.677	0.458		
**Behavior (BEH)**	BEH1: The film-watching experience of preview films during this summer season.	0.722	0.521	0.782	Chen et al., 2017; Wang, 2023 [24, 43]
	BEH2: My past years’ experience of purchasing tickets and watching preview films.	0.670	0.449		
	BEH3: The frequency of attending preview screenings has increased in recent years.	0.819	0.671		

Convergent validity and discriminant validity were used to examine the validity of the measurement scale. Convergent validity measures the amount of shared variance among the variables. It was assessed by the Average Variance Extracted (AVE) value. According to Fornell and Larcker [42], an AVE should not be lower than 0.4 to demonstrate an acceptable level of convergent validity. The analysis revealed good convergent validity across different dimensions of the scale. Discriminant validity was examined using Pearson’s correlation coefficient. In Table 2, the square root of the AVE values (shown in bold along the diagonal) is greater than the Pearson correlations among the variables. Hence, it confirms good discriminant validity.

**Table 2:** Validity analysis of scale.

Variables	Converge	Pearson Correlation Coefficient and Discriminant				
	AVE	Perceived behavioral control	Consumption expectation	Social network	Intention	Behavior
**Perceived behavioral control**	0.464	**0.681**				
**Consumption expectation**	0.484	0.194	**0.696**			
**Social network**	0.429	0.242	0.267	**0.655**		
**Intention**	0.430	0.247	0.200	0.254	**0.656**	
**Behavior**	0.547	0.195	0.224	0.241	0.231	**0.740**

#### 4.2.2 Normality and multicollinearity test

First, we tested the normal distribution of the data. In a standard normal distribution, the data should be distributed around a center where the mean is 0. However, in most questionnaire surveys, the data is often difficult to follow the standard normal distribution, but can only approximate the requirements. We believe that the approximation still satisfies the normal hypothesis. During data processing of structural equation model, normality test was carried out to ensure that the variables in structural equation meet the requirements. Among them, using skewness and kurtosis to test the normality of questionnaire data is a common method. In this study, we improved the rigor of normality test and adopted the judging method based on the skewness value and the kurtosis value. When the absolute value of the skewness becomes less than 3 and the absolute value of the kurtosis value becomes less than 10, we believe that the data follows a normal distribution and that it supports subsequent data analysis. Here, we inserted the table of skewness and kurtosis test to show the skewness and kurtosis of the data. Through optimization and refinement, we made the test procedure clearer and more rigorous. Further data analysis and model building can be made. Table 3 shows the normal distribution test.

**Table 3:** The normal distribution test.

	N	minimal value	maximum value	mean value	standard deviation	skewness		kurtosis	
	statistics	statistics	statistics	statistics	statistics	statistics	standard error	statistics	standard error
**PBC1**	251	2	7	5.43	.842	-.697	.154	1.305	.306
**PBC2**	251	3	7	5.64	.942	-.287	.154	-.287	.306
**PBC3**	251	2	7	5.47	.926	-.734	.154	.799	.306
**CE1**	251	2	7	5.29	.800	-.724	.154	.808	.306
**CE2**	251	3	7	5.43	.894	-.156	.154	-.183	.306
**CE3**	251	2	7	5.45	.863	-.699	.154	1.126	.306
**SN1**	251	3	7	5.74	.909	-.550	.154	.162	.306
**SN2**	251	3	7	5.59	.841	-.326	.154	.131	.306
**SN3**	251	2	7	5.67	.902	-.516	.154	.561	.306
**INT1**	251	3	7	5.50	.817	-.229	.154	.168	.306
**INT2**	251	3	7	5.60	.926	-.426	.154	-.021	.306
**INT3**	251	3	7	5.63	.904	-.388	.154	.134	.306
**BEH1**	251	2	7	5.25	.793	-1.111	.154	1.922	.306
**BEH2**	251	2	7	5.45	.943	-.866	.154	1.284	.306
**BEH3**	251	2	7	5.18	.835	-.714	.154	.756	.306

Secondly, we added a multicollinearity test to investigate how well one independent variable can be represented by the other independent variables. We took behavior as the dependent variable and other variables as independent variables, and carried out collinear diagnosis between variables. Variance inflation factor (VIF) was used to detect the severity of multicollinearity in the analysis. When the value of VIF is greater than 5, there is probably a collinearity problem. When the VIF value is greater than 10, the collinearity problem is regarded as serious. After analyzing the VIF values, we found that VIF values were all smaller than 5. In other words, there was no collinearity problem between our variables. The results are shown in Table 4.

**Table 4:** Multicollinearity test.

Model	Nonnormalized coefficient		Standardization coefficient	t	Sig.	Collinear statistics	
	B	standard error	trial version			tolerance	VIF
**Constant**	.712	.347		2.054	.041		
**CE**	.273	.064	.263	4.290	.000	.624	1.603
**SN**	.184	.064	.179	2.860	.005	.599	1.671
**PBC**	.117	.060	.119	1.952	.052	.635	1.575
**INT**	.254	.064	.250	3.948	.000	.586	1.708

#### 4.2.3 Hypothesis testing

The model’s path analysis and evaluation were carried out using the AMOS 22.0. The results demonstrate an outstanding model fit, as indicated by CMIN/DF, RMSEA, GIF, AGIF, CFI and NFI. CMIN/DF represents degrees of freedom divided by chi-square. The smaller the indicator, the better the model fits the data. A CMIN/DF value fewer than five typically indicates that the model matches the data well. "Root Mean Square Error of Approximation" (RMSEA) is another statistical metric used to quantify the degree of model fit in structural equation models. The RMSEA index shows both the mean and variance of the fitting index. If RMSEA is larger than 0.08, the model has a poor fit. Additional factors include the Goodness of Fit Index (GFI), Adjusted Goodness of Fit Index (AGFI), Comparative Fit Index (CFI), and Normalized Fit Index (NFI). The higher the model’s degree of fitting, the bigger the values of these parameters. Table 5 shows the main fit indices of the model [41].

**Table 5:** The main fit indices of the model.

Fit indices	CMIN/DF	RMSEA	GIF	AGIF	CFI	NFI
**Standard values**	<5	<0.080	>0.800	>0.800	>0.900	>0.800
**Measurement values**	2.100	0.066	0.932	0.912	0.900	0.875

According to the results of hypothesis tests, the variables of perceived behavioral control, consumer attitude, and social network can explain 75% of the variance in the research model, which reflects consumers’ intention to watch films under the influence of large-scale preview screenings. The model can explain 64% of the variance in consumers’ actual film-watching behavior. These results demonstrate the research model’s strong explanatory capacity. All variables in the model had significant positive influences on the dependent variable. Table 6 summarizes all the hypothesis testing results.

**Table 6:** Summary of hypotheses test results.

Hypotheses	Paths	whether support or not
**H1**	Consumption expectation → Intention	Support
**H2**	Social network → Intention	Support
**H3**	Perceived behavioral control → Intention	Support
**H4**	Intention → Behavior	Support

Of all the influencing factors, perceived behavioral control has the most impact on the willingness of consumers to attend large-scale preview screenings. The significance level p is less than 0.001, while the path coefficient of perceived behavioral control β is 0.409. Large-scale preview screenings are affected by consumer’s perceived behavior control, which is a complex process that involves the consumers’ perceptions of the difficulty of go to the cinema and how these perceptions affect their decisions. During the large-scale preview screenings, consumers often experience information asymmetry and encounter unclear issues. Their perceptions of the films are primarily based on film trailers and advertisement. As a result, during this stage, consumers will focus more on their own perception to decide whether to watch the film in advance. This control over behavior is evident in factors like knowing the director’s previous work, whether there are difficulties to watch the film, whether early viewing will cost more than regular viewing. This viewpoint is in line with that of Ai, who thinks that obstacles should be eliminated to encourage people to attend the screenings in Chinese film market [45]. Perceived behavioral control evaluates an individual’s belief and ability to perform a behavior. It pertains to the individual’s confidence in their ability to overcome challenges and achieve goals. Based on movie-watching behavior, perceived behavioral control includes the audience’s access to movie information, the audience’s experiences of movie watching, and the personal viewing capability. This study found that perceived behavioral control directly influences the audience’s motivation to watch blockbusters. When viewers perceive that they can and it is convenient to reach the preferred movie resources, they are more inclined and ready to watch films. Conversely, if the audience thinks there is a barrier that prevent them watching films, they will be less willing to go to the cinema. Prior research indicates that perceptual behavior control consistently affects movie-watching habits across different cultural backgrounds. The selection of films is influenced by analogous psychological elements. Even though people from different cultures have different tastes in movies and different ideas about what looks good, perceptual behavior control has a big impact on their viewing behavior.

According to the findings, the social network’s path coefficient (β) is 0.300, meaning that it is significant at the p-value threshold of less than 0.05. Social network has become another significant factor that influence consumers’ willingness to watch films because they can provide social support for consumers’ knowledge asymmetry at the large-scale preview screenings. Within a given community, community norms, word-of-mouth and professional, family, and friend recommendations can affect the reviewers’ decisions about watching movies. Even though there is a knowledge asymmetry during the movie-viewing, the community attribute can offer significant support. A positive word-of-mouth can be created by recommendations and evaluations from community members who are opinion leaders and devoted followers, as they play a significant role in the knowledge diffusion [20, 46]. Group influences and social norms frequently accompany mass viewership. Customers will feel more willing to watch movies if they believe that others are actively doing so or if friends and they are invited or recommended by family members to do so. In addition, consumers will be more likely to watch movies if they believe that engaging in preview screening is trendy. Consequently, recommendations from friends, family members, and professionals as well as positive word-of-mouth can directly affect consumers’ motivation to watch movies.

The findings demonstrate that consumers’ willingness to attend a large-scale preview screening is significantly affected by their consumption expectation. This influencing factor’s path coefficient (β) in the model is 0.272, and the p-value is at the 0.05 level of significance. The demands of consumers on movie-watching are growing as the theater is upgraded and projection technology is refined. The overall sound experience, images and culture become key factors that influence people’s decision to watch movies ahead of time. While perceived behavioral control and social network can greatly affect consumers’ intention to watch films, consumption expectation remains a significant determinant. During the preview stage, customers may decide to watch the film ahead of time due to their own consumption expectations and trust. Consumer satisfaction will increase if they have a great viewing experience during a large-scale preview screening. Such experience can be affected by a comfortable setting, excellent audio-visual quality, stories worth watching, etc. Because of enjoyable viewing experience, consumers will be more likely to participate in such early preview screenings. This factor’s impact is similar to that of Habes et al., who discovered that expectation has a significant influence on viewers’ YouTube video selection [47]. Thanks to developments in projection technology and theater hardware, consumers now care more about the audio-visual experience and a theater’s ambience when they enter. Consumer expectations about consumption and their overall viewing experience will affect their inclination to attend large-scale preview screenings.

**Fig 6:**
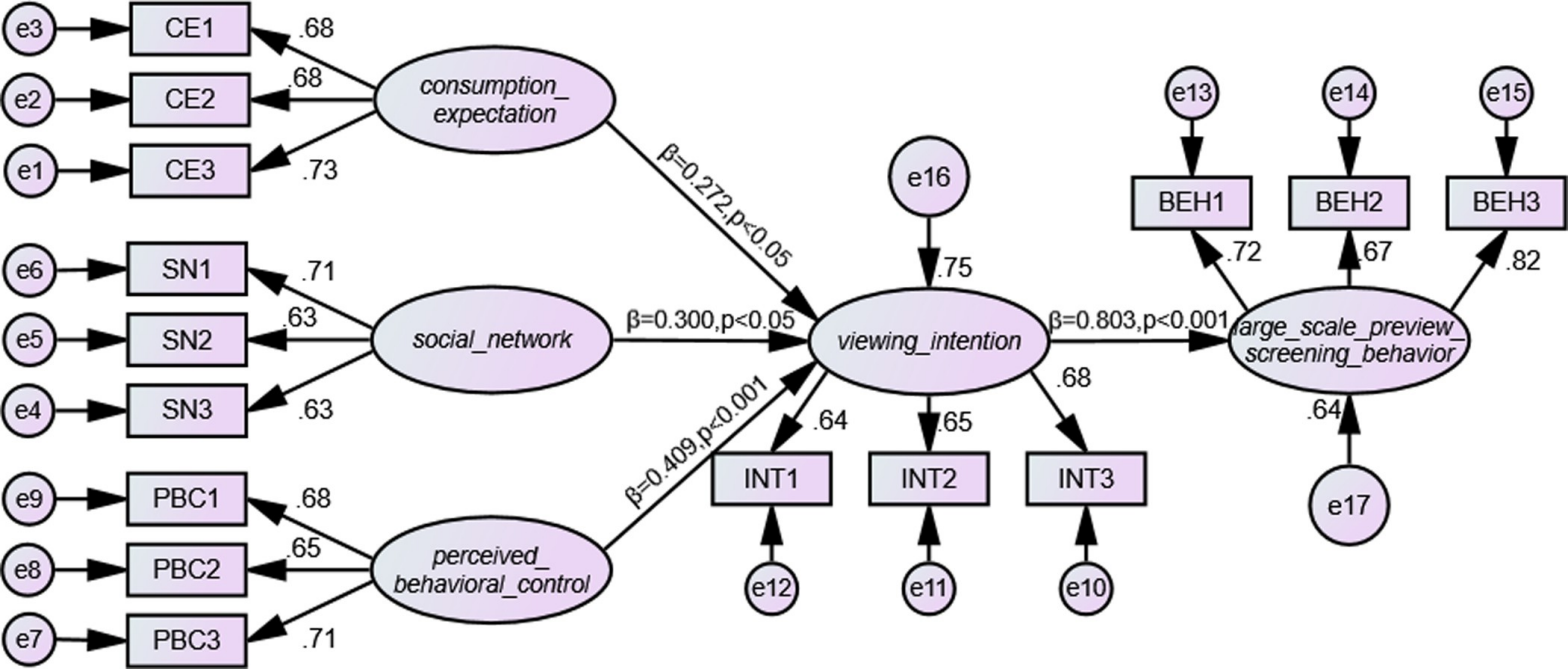
Analysis of model paths.

There is a direct and positive correlation between consumers’ intention to watch large-scale preview screenings and their actual viewing behavior. The findings demonstrate that large-scale preview screening behavior is significantly affected by consumers’ intention, which is indicated by a p-value of 0.001. The large-scale preview screening behavior’s route influence coefficient (β) is 0.803. Consumers’ willingness to watch movies in large preview screenings can best explain their behavior. It shows how growing consumer demand for movies might translate into bigger box office receipts for high-budget on-demand projects. A consistent flow of revenue can be generated to support the film industry’s rapid and healthy expansion. The findings of the path analysis are displayed in Fig 6. The substantial correlation between the two variables of intention can be confirmed by other studies on consumption behavior, even though there aren’t many studies specifically examining large-scale viewing behavior. For example, customers’ behavior and their inclination to purchase environmental-friendly products were found to be positively correlated by researchers [48, 49].

## 5. Conclusions and recommendations

This study has examined the factors that influence consumers’ intention to watch films on large-scale preview screenings. It carried out an offline survey on 251 consumers in Zhengzhou, a new first-tier city in China. The TPB theory was integrated with a newly built model. This model successfully reflected factors that influence consumers’ willingness to watch films and their behavior under large-scale viewing. What’s more, an exogenous variable of consumption expectation was added to the model to broaden its application. In this study, consumer perceived behavior control and social network were taken as two essential variables. The study’s findings indicate that consumers’ intention to watch films are significantly influenced by their perceptions of their own behavior control, subjective standards, and consumption expectations. Social network and consumption expectations are the next most important factors that influence consumers’ large-scale preview screenings, ranking next to the perceptual behavior control. Through this study, we have discovered the pattern of consumer movie-watching behavior and discussed its positive impact on the sound development of Chinese film market. This paper can be used to understand the intention and behavior of moviegoers in first-tier cities. They can also be used as a reference by producers to improve marketing strategies and by theatres to improve their services. This research helps identify the core movie-watching behavior rule underlying a certain movie-watching phenomenon among first-tier city customers. It will help boost moviegoers’ passion, increase Chinese film industry box office revenues, and support the film market’s growth.

This research provides insights for all stakeholders in the large-scale preview screening sector. First, to attain widespread viewership, a film producer must enhance the film’s quality and storyline by strategically using the intellectual property (IP), thereby winning consumer perception and consumption recognition. Due to their extended life cycle, high-quality and the state-of-the-art screenings enable consumers to get the best experience. During the preview screening state, producers can let word of mouth take over as moviegoers spread their opinions about movies through social networks to enhance the film’s appeal is enhanced. Secondly, cinemas should enhance their amenities, including lighting, ergonomic seating, and services to optimize consumer experience during film viewings. Cinemas must enhance their film scheduling tactics to improve consumers’ movie experience during both regular releases and preview shows. They must guarantee that the preview screening strategy does not adversely affect the box office of released films. By adjusting the dates and venues for preview screenings, they can satisfy audience demand and optimize available resources. Third, film distributors or marketers can optimize the benefits of screening based on film attributes and market demand by establishing word-of-mouth marketing, gathering market input, fine-tuning the screening size, maximizing screening time, and bolstering social media awareness. Fourth, film authorities and industry associations should establish norms and restrictions on mass screenings. To foster the robust growth of the film industry, regulators must conduct additional research and establish pertinent rules to encourage the sustainable development of China’s film sector and mitigate predatory competing practices.

This study has some limitations. By the year of 2023, preview screenings have become a popular strategy in the Chinese film industry. However, it may be still rare in other countries’ film markets. Large-scale moviegoers have distinctive traits, we focused on representative consumers from China’s typical first-tier cities. During the sample selection, the new first-tier cities were chosen to reflect the average standard of Chinese film market. Future studies can expand the sample size by selecting other kinds of urban moviegoers for comparison.
